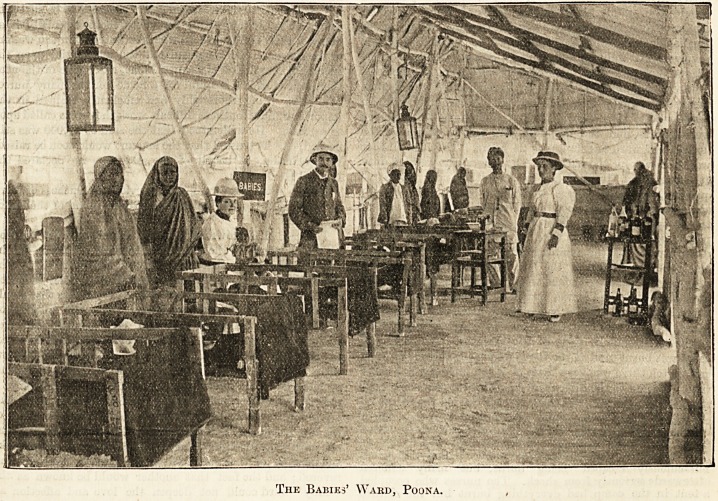# "The Hospital" Nursing Mirror

**Published:** 1899-07-22

**Authors:** 


### "The Hospital" Nursing Mirror.

Being the Nursing Section of "The Hospital."

[Contributions for this Section of "The Hospital" should be addressed to the Editor, The Hospital, 28 & 29, Southampton Street, Strand, London, W.C., and should have the word "Nursing" plainly written in left-hand top corner of the envelope.]

### Notes on News from the Nursing World.

### THE MARLBOROUGH HOUSE RECEPTION ON FRIDAY.

Those nurses who are unable to attend the Marlborough House ceremony—we hope the number will be small—can have their certificates sent to them by post on application by letter to the office of the Royal National Pension Fund for Nurses, 28, Finsbury Pavement, E.C., enclosing a penny stamp and quoting their policy number.

### THE PRINCE OF WALES'S SURPRISE VISIT.

It is not often that Royal personages pay surprise visits in this country. There is an idea, too, among people who know little of their habits, that because they late take rest they do not rise early. If the appearance of the Prince of Wales at ten a.m. on Monday morning caused a flutter of excitement in the wards of the Princess Alice Hospital at Eastbourne, it also, doubtless, created equal astonishment to many of the townsfolks. Fortunately, a hospital is not like some private houses, and the Prince found everything in as apple-pie order as if his visit had been announced beforehand, though it is possible that a few of the nurses felt regret that they had not a chance of putting on their smartest cap for the occasion.

### A FORTUNATE YOUNG COUPLE.

When the Duke and Duchess of York visited Queen Charlotte's Hospital on Monday to open the new nurses' home they took the opportunity of inspecting the hospital itself. Everything looked very bright and dainty, from the pupil nurses in their white frocks to the small morsels of humanity which they displayed with such care and pride. A pair of twin babies proved a great attraction to the guests. They were so much alike that it would have taken the discerning glance of mother or nurse to distinguish the one from the other, had not the boy worn a dark red ribbon, and the girl a blue one. Their Royal Highnesses made a point of going to see them, so that in years to come when "George" and "May" arrive at years of discretion, they will be able to triumph over less lucky boys and girls, because not only have they been named after the Duke and Duchess, but they can boast that they were presented to Royalty within the first week of their existence.

### HOSPITALS IN LUCK.

The Earl of Leicester has afforded yet another practical proof of the interest he takes in the fine charitable institutions in his county. He has sent a donation of £5,000 to the governors of the Norfolk and Norwich Hospital for the purpose of erecting a nurses' home. This makes no less then £40,000 which he has given to the hospital on different occasions. The latest donation will not be the least appreciated, for a nurses' home has long been badly wanted at Norwich. The other fortunate hospital is the Royal Alexandra, Rhyl, to which the Duke of Westminster has presented the munificent sum of £10,000. This amount was won by his horse Flying Fox last week, and the gift will enable the committee to commence the erection of the administration block at once.

### MIDWIFERY TRAINING.

On Tuesday the Lord President of the Council received a deputation on the question of legislation for midwives. The deputation was introduced by Mr. Heywood Johnstone, M.P., and amongst the societies represented were the London Obstetrical Society, Queen Victoria's Jubilee Institute, Women's Liberal Federation, Women's National Liberal Association, Women's Liberal Unionist Association, Women's Cooperative Guild, Women's Industrial Council, National Union of Women Workers, and the Midwives' Institute. After hearing several of a deputation, the Duke of Devonshire promised to try and induce the Cabinet to take up the Midwives' Registration Bill another session. A medical practitioner in an outlying suburb of the metropolis remarked the other day that numbers of maternity nurses ask him for work. His first question is, "Are you qualified?" and in reply they display important parchments stating that they have been trained at a particular hospital for three months or six weeks, as the case may be. His next question, "Can you make beef tea or a custard pudding?" as often as not elicits the answer, "No, that is the cook's work." "What are your terms?" he then inquires, and the answer usually is, "Eight to ten guineas the month"; and, adds our informant, "they cannot nurse either." This is a state of things that requires to be altered.

### CHELSEA INFIRMARY.

We are glad to learn that Miss de Pledge's scheme for a reorganisation of the nursing arrangements at the Chelsea Infirmary, especially with regard to a readjustment of salaries on a more up-to-date basis, was unanimously approved of by the Board of Guardians at their last meeting. Her further recommendation that four additional charge nurses should be appointed, was also adopted without discussion. This increase to the staff will allow of a fully qualified head to each ward instead of one to two wards as has hitherto been the case, and cannot but make for more efficient administration all round. Miss de Pledge and the guardians are to be congratulated on the improvements thus effected in the nursing department.

### NURSES IN THE HAY-FIELD.

A very pleasing example has been set by the President of the Devon and Exeter Hospital, Lieutenant-Colonel Lucas, and his daughters, who last week entertained the whole of the nursing staff at Dunchidcock House, their beautiful Devonshire residence at the foot of the Haldon Hills. Colonel Lucas sent two brakes on Monday, which conveyed the sisters, night nurses, and as many probationers as could be spared, and on Wednesday the two brakes were again filled with the staff nurses, the rest of the probationers, and the assistant matron. The drive out was greatly enjoyed in lovely weather, and as each party arrived they were conducted by the Misses Lucas to the fruit gardens, there to help themselves *ad lib.* Then they proceeded to the hay-field, and it was a joyous sight to watch their happy faces whilst busily making hay, riding in the hay-carts, and some resting on hay-pillows in the shade. Tea, with fruit and cream, was provided under the trees, and most heartily enjoyed by everyone. Subsequently, a stroll was taken through picturesque scenery to the pretty little thirteenth-century church, which is well worth seeing, one special feature being the beautiful and finely-carved oak screen. The party then prepared for the drive home, with plenty of flowers, gratitude, and sweet recollections of much kindness and a very happy day.

### A CURIOUS CONCEPTION OF THE NURSING PROFESSION.

It is stated in a journal which is supposed to devote itself to the interests of women, that the friends of Lady Hermione Blackwood "rather fear" the result of her admission to the London Hospital. Our contemporary avers, in tearful tones, that the hospital is "in the worst quarter of London"; and adds, in a horrified manner, that some of the sights Lady Hermione will encounter "are too dreadful for description." We do not believe for a moment that this sort of rubbish meets with the approval of Lady Hermione, or of Lord and Lady Dufferin. The sights the former will encounter are encountered by any number of ladies as refined and carefully nurtured as even the daughter of a distinguished peer. No one who shrinks from witnessing the most painful spectacles should dream of joining the nursing profession. A soldier who wanted to run away would be of little use on the field of battle; and a nurse who wishes to pick and choose her patients has mistaken her vocation.

### THE NURSES OF THE PRESTON ROYAL INFIRMARY.

The annual outing of the nurses and staff of this infirmary was provided this week by the generosity of Dr. R. C. Brown, one of the honorary doctors. He has for years kindly given a treat to the nurses, in order to make them forget for a short time the many sad surroundings of suffering and the constant strain they have to bear, by a trip to the seaside. Early in the morning all awaited the conveyance to take the party to the Central Station, *en route* for Fleetwood. After exploring the town a rush was made for the boats, in order that a couple of hours on the sea might bring the lost colour to many cheeks. The sea being calm, all thoroughly enjoyed themselves in viewing the picturesque bay of Morecambe, with its iron lighthouse in mid-channel. Dinner was provided at the Station Hotel, and the party left by the new electric light railway for Blackpool. Here the entertainment was varied by a drive round some of the environs, and many had the pleasure of a promenade on the North Pier. Tea was partaken of at the Winter Gardens, a highly satisfactory ending to the day being brought about by a call at the Grand Theatre.

### BOYCOTTING THE MODERN NURSE.

A medical man, whose name is not given, is allowed to indulge a strong attack upon nurses in a medical journal. In the course of his letter, he says, "Two so-called 'lady' nurses were introduced into this district a little over a year ago, and have made themselves such a nuisance that nearly six months since I intimated to the nursing committee that I could not allow them to come to my patients, and told my patients that if they had the nurses in their houses I would not attend them." This anonymous correspondent does not hint at the locality of the district to which he alludes, but we can hardly believe that his statement that his "firm attitude" has not caused his practice to suffer in any way will long continue to be true. We commend to his attention the different attitude of Dr. F. A. Taylor and Dr. E. Buckell, of Romsey, who, after a year's experience of the work done by the nurses employed by the Romsey Association, are generously giving their services free to all patients admitted to the new Nursing Home opened by Princess Henry of Battenberg last January. But, of course, the attitude of the medical profession generally is not to boycott, but to welcome the modern nurse.

### DISTRESSING EXPERIENCE OF A NURSE.

The temperature of young children is not, or at least should not be, taken through the mouth. But no danger of accident is anticipated in the case of grown-up people, and the circumstances of the death of Mr. James McAskie on Monday are very curious. While the nurse in charge of him was taking his temperature he bit off part of the clinical thermometer which she had placed in his mouth, and swallowed some of the glass and mercury. He had been through an operation for abscess of the kidneys, and was under the influence of a morphia injection at the time. No blame, apparently, attaches to the nurse, but the deplorable incident seems to point to the desirability, in similar conditions, of taking the temperature in another manner.

### FRICTION BETWEEN THE MATRON AND THE NURSE AT DEVONPORT INFIRMARY.

The Infirmary Committee of the Devonport Board of Guardians in an exhaustive report on the nursing staff and the management and general condition of the infirmary suggest that the Local Government Board should be urged to issue an order directing that matrons of workhouses should have no jurisdiction in infirmaries, but that the superintendent nurse should exercise paramount authority. In this particular case they found that the friction between the matron and the superintendent nurse, of which a great deal has been heard in the locality, arose through the latter holding the reins a little tighter than they had heretofore been held, and the manifestation of her desire and determination to put everything in the infirmary without delay on an up-to-date basis. But they do not think that she was more strict or zealous than she ought to be; and in many matters of detail, such as the cooking for the nurses, attention to their rooms, and the supply of linen, they clearly entirely agree with her. There is little doubt that by degrees the Boards of Guardians will come round to the opinion that the best arrangement for all parties would be to give the superintendent nurse in the infirmary full authority and control, subject only to the supervision of the medical officer.

### NEATH NURSING CHARITY.

After two years of life this charity appears to be immensely appreciated in the Welsh town. In the report for 1898-9 just issued it is stated that besides the people in the homes of the very poor the nurse—who paid 3,071 visits in 12 months—has numerous patients among the artisan class, who have sufficient means to provide the necessary food and clothing for the invalid, but where relatives in extreme illness lack the skill and the knowledge acquired by a trained nurse. Another point is that, "In all cases the nurse works entirely in unison with the doctors, not taking the responsibility on herself, but endeavouring to carry out their wishes for the patients." This is as it should be; and it is also satisfactory that Miss Franks (one of the inspectors of Queen's Nurses) inspected the branch at the beginning of the year, visited some of the cases under treatment, examined the books of the affiliated charity, and declared herself well pleased with the work at Neath.

### THE CASE OF NURSE CAVARET.

The members of the Metropolitan Asylums Board are to be congratulated upon their decision in reference to the hard case of Miss Cavaret, which was mentioned in this column a fortnight ago. On further consideration of the matter, the committee recommended that she should be awarded £1 per week for 12 months, at the end of which time they would report to the Board the state of her health. This is more liberal treatment than was originally proposed, and it may be taken for granted that the sanction of the Local Government Board will be given. It may, however, be hoped that, profiting by the experience of Nurse Cavaret, young nurses entering the public service in future will not decline to avail themselves of the provisions of the Poor Law Officers' Superannuation Act of 1896.

### NURSES IN YUKON.

Some interesting details are given in a Montreal paper by Miss Georgina Powell, of the Victorian Order of Nurses, who is working as the superintendent of the Order in the Yukon. Miss Powell states that the sanitary condition of Dawson is being looked after, and that the people are obliged to keep their premises clean and tidy. In the Good Samaritan Hospital there were 20 patients at the time she wrote under the care of Nurse Hanna. Of the latter's devotion she speaks in the highest terms, and says, "She is so tired when she comes home at night that she flings herself down anywhere and sleeps." Unfortunately, eight cases of typhoid fever have occurred in Dawson. These have been admitted to St. Mary's Hospital; now, as Nurse Powell adds, "our work is coming on." The Government officials do all they can to help the nurses, and quite look upon them as a part of their own staff.

### A NURSING HOME FOR LICHFIELD.

On Monday there will be opened at Lichfield a Nursing Home of suitable dimensions. It will be a memorial of the 1887 and 1897 Jubilees of Queen Victoria. The Executive Committee have been assisted in their work of preparation by Miss Scott, Miss Graham, and Nurse Harding. Archdeacon Scott was the originator of the scheme, and it is a matter of much regret that he has not lived to see the fulfilment of it.

### WANTED-A MEDICAL MISSION.

The value of nursing work in the mission field was pointed out the other day at the anniversary of the Universities Mission to Central Africa. The Bishop of Zanzibar is very anxious to establish a medical mission in Pemba, an island off the East African coast. The establishment is to consist of nurses and lady workers, to work amongst the women on medical and nursing lines. We assume that the "lady workers" will be qualified medical practitioners. A house has been built by the Sultan's Government, some nurses have volunteered, but, alas! funds are wanting. Yet "there is no doubt," said a speaker at the meeting, "that you can reach through medical channels and the instrumentality of nurses large bodies of people . . . . impervious to theological argument."

### NURSING AT A MILITARY HOSPITAL IN THE MAURITIUS.

The nurse in charge of the female department of the Military Hospital at Curepipe Camp, Mauritius, who is working under the local committee of the Soldiers and Sailors' Families Association, sends us a few interesting particulars. After spending ten days in quarantine because the vessel had called at an infected port—Mauritius itself is now in quarantine—she arrived on December 28th last. Curepipe, she states, is a very nice place, except when it rains, "which is nearly always." It is the healthiest spot on the island. The camp is two miles from the village, in which the shops are kept chiefly by Arabs and Chinese. The latter call themselves "consolidated retailers," and the odour from some of their establishments is unpleasantly pungent. The servants are Creoles and Indians, most of them being men. The hospital consists of only two rooms set apart in the married quarters; but, as there are very few soldiers' wives, the nurse finds it sufficient. Her rooms adjoin, and together with the hospital are shut in by a gate from the rest of the block. She has had five patients—maternity cases—in four months. A native servant is allowed her, but she goes away at night, and comes again in the morning. The salary she describes as "a very good one," but as everything except fruit and labour is much more expensive than in England, she does not consider that she is much richer.

### SHORT ITEMS.

The Queen will open the Diamond Jubilee Wing of the Isle of Wight Infirmary at Ryde on Friday, the 28th.—Mr. Chamberlain will attend the annual meeting of the Colonial Nursing Association at Stafford House on Tuesday afternoon.—The Mayor of Bradford has presented a gold medal to Nurse Vickars and Nurse Farquharson respectively, for having obtained the highest number of marks in the recent examinations in surgical and medical nursing. The two nurses were also each presented with a book.—The seventh annual meeting of the first Home established under the auspices of the Women's Convalescent Home Association which is in a most favourable position at South Linford, was held on Saturday. The report stated that during the year the stay at Sea View averaged a fortnight for 700 patients.

### Gynæcological Nursing.

By G. A. Hawkins-Ambler, F.R.C.S., Surgeon to the Samaritan Free Hospital for Women; Assistant Surgeon to the Stanley Hospital, Liverpool.

(Continued from page 206.)

### DRESSING OF PATIENTS.

For the ordinary dressing of patients the following instruments are generally required: Sims's speculum, Fergusson's speculum, uterine hook, vulcellum forceps, uterine dressing forceps, uterine sound, several Playfair's probes, uterine scarifier, Neugebaur's speculum, mop holders prepared with dabs of absorbent wool, or forceps ready charged with wool mops. In addition, some use different special instruments. I, for example, use my uterine screw probes for applying various dressings to the uterus; others use gauze applicators or forceps for the application of gauze; but the above represent what is ordinarily required for the dressing of a series of cases. Besides which you require a supply of absorbent wool (plain, sterilised, or antiseptic), sal alembroth or double cyanide gauze, tampons ready prepared, and such medications as commend themselves to the practitioner. These are usually glycerine, ichthyol and glycerine, with which to soak tampons, nitric acid, iodised phenol, nitrate of silver, and so forth. The Playfair's probes should be ready covered with a thin, smooth layer of absorbent wool. This is done by laying a thin layer of wool on your left palm. Place your probe, damped with water, in the inner edge of it, and roll it outwards, thus wrapping the wool closely and firmly round it. Where such a caustic as nitric acid is to be used the probe will be made of aluminium, and the application will be made through a glass Fergusson's speculum, as the chemical would have a corroding effect on metal.

It is essential that your instruments be clean and sterilised. In rapid succession of cases it is impossible to secure this, but you must thoroughly scrub each one after use with soap and water. In the case of the uterine sound it is necessary to be extremely particular. Warm it before use by dipping in a hot solution of carbolic acid and water, 1 in 20 or 40. After using wash thoroughly, and if there are no facilities for boiling it run the end of it once or twice through the flame of a spirit lamp, which is a good disinfectant. It is wise to familiarise yourself with the names of various instruments in use for gynæcological work. All the instruments must be kept perfectly clean and in good order. Cracked or broken specula, for example, must not be used. Different surgeons have a fondness for different forms of the same instrument. The best instrument is the one that a man can use best, and what would be clumsy to one is a valuable instrument to another. Surgeons vary, too, as to the extent of their manipulations in the out-patient room or in their consulting-room, and while some men are content with two or three instruments of the simplest possible design, others can work best with an elaborate series of appliances. An ordinary surgical dressing case is a necessary adjunct to your collection, with its scissors, artery forceps, knife, &c.

You will be expected not only to hand the instrument asked for, but to pass it ready for use. Have hot water or carbolic lotion ready for warming instruments of all kinds—it is cleanlier than the hand; when passing a speculum pass it warmed, and lubricated on the outside, clean and highly polished. Do not drop drugs about the place, or supply them or the lubricants and dressings dripping with messy applications to annoy the surgeon or dirty the patient's clothes. Often in holding a Sims's speculum you can raise the uppermost buttock, and if the hair on the genitals be thick this might be pulled out of the way, so giving a great deal more light at times. A little observation and consideration will make you quite familiar with the surgeon's methods, and you will be able in a surprising degree to anticipate his wishes with the instrument he requires, and be not only a third hand, but a helpful brain to him, saving a vast amount of time, making his work infinitely simpler and more effective, and preventing a good deal of irritability which comes to the man who has to worry about directing every movement of his assistant.

### Tampons and Plugs.

Applications are often made to the vagina by means of plugs or tampons. These are readily made by tying a piece of silk or new string five or six inches in length round a small piece of absorbent wool. The wool will vary in size, being usually loosely folded to the size of a bantam's egg, and sometimes two or three tampons are attached to the same piece of string like a kite's tail. Or absorbent wool may be wrapped in gauze, and the pear-shaped mass fixed to a piece of string or silk, attached to the thinner extremity.

A tampon before use is well soaked with glycerine, or with glycerine and ichthyol, 5 to 15 per cent., or whatever medication we desire to apply, and the next business is to transfer it to the vagina. This can be done in several ways. Very often it is sufficient to bring the patient to the edge of the bed, make her lie in the Sims position, and holding a prepared tampon in the right hand, pass the left forefinger into a lax vagina and pull back the posterior wall very steadily. You will then, in most cases, be able to slip the tampon inside, and press it up with your finger in the direction indicated by the surgeon. Some nurses use a tampon-introducer, but most of them find it more difficult to insert than the plain tampon. Another way of inserting them is by passing the small blade of a Sims speculum, the back of which has been well greased, into the vagina. Press the greased portion against the posterior part of the orifice, and it will gently slide in. You will now pull it back sufficiently to admit the tampon or plug, which can be pressed along the groove of the instrument with the right forefinger, while with the left hand you withdraw the speculum. It may also be introduced through a Ferguson's cylindrical speculum, which is a long tube, black on the outside, and with a polished inner surface. Its lower end is rimmed, and the upper end is narrower and uneven, the posterior part longer than the anterior. This posterior part is pressed against the posterior wall of the vagina, which is gently bent back by the forefinger, and with a little steady pressure the speculum, which has been already lubricated, passes in. You can now push the tampon along it, but you will have to use a clean, straight instrument of some sort (forceps) to hold it in position while you withdraw the speculum, which is longer than the finger. After the tampon has been introduced, it will be necessary in most cases for the patient to wear a diaper, since these applications frequently cause a considerable discharge. The tampon may be removed in twelve hours.

Where a patient is to be plugged for hæmorrhage, it will usually be done by the doctor himself, though in an emergency it is just as well for a nurse to know how to do it. She will introduce a Sims speculum after preparing a number of small plugs of absorbent wool (these will be better wrung out of water), and she must then systematically pack these in the vagina, filling the top of the vagina and every portion of it with the plugs, to each of which a string is attached for its subsequent withdrawal. Unless this is done very carefully, it will not be of any use at all, and if done once thoroughly, it is a very effectual way of stopping hæmorrhage. If the plugs have been wrung out of carbolic lotion, one in forty, it will be all the better, and a diaper or pad may be placed on the vulva—the external genitals—and kept firmly in position by a T bandage. It can also be done by passing a piece of lint into the vagina, and packing the plugs into this, which makes it more easy to withdraw.

### A Year's Plague Nursing in India.

By a Sister.

INOCULATION AND OTHER EXPERIENCES.

I had heard that the effect of inoculation was pretty quick and that you began to feel ill about two hours afterwards, so as it happened to be mail day I got my letters written in good time, leaving a small space to fill in telling them at home how I was feeling after inoculation. Well, we proceeded to the General Hospital, where we met the doctor and a qualified native assistant, and after the usual preliminary cleansing of the skin with carbolic, I had 5 cc. of Professor Haffkin's "preventive serum" injected through a glass syringe with a hypodermic needle. So many people have asked me whether it is like vaccination, but of course it is not, as the serum is injected subcutaneously and a far larger quantity used. This resulted in a fair-sized swelling, and before very long my arm became red and swollen down to the elbow with a general feeling of stiffness in the axilla. A large area round the place of inoculation also felt very tense and hard; that was, of course, not pleasant, but as two hours passed away without any further feeling of discomfort I was able to add cheerfully to my letters that I was all right. However, later on towards the evening I began to feel feverish and had a temperature of about 100 deg., and was glad to retire to bed early, though except for a feeling of soreness I really did not feel ill. My temperature next morning was 99 deg., and the local symptoms were about the same. This continued for about a week, and gradually the swelling dispersed, but I felt very much pulled down, almost as though I had just recovered from an attack of influenza so I had to take a tonic for a few days which soon set me right. Some of the nurses who were inoculated were affected in different ways, in one case there was a decided rash over the arm. The actual local effect was about the same in all cases. In order to be thoroughly free from peril it is necessary to be inoculated every six months.

The Babies' Ward, Poona.

About February plague began to decrease very rapidly in Poona, and we soon had far easier times and were able to pay more individual attention to our patients, which was better for them and more interesting for us. The cases in the various wards were of an extremely severe form, but some had made marvellous recoveries. An interesting case was that of a woman whose symptoms were complicated with hemiplegia and whom we had great difficulty in nursing. Often, indeed, we despaired of her life. The difficulty in keeping her free from bedsores was very great owing to her wasted condition, but I am glad to say we eventually saw her well and happy. She regained her speech and was able to walk out of the hospital with little trouble. Sometimes we had cases of very troublesome synovitis. These were very obstinate and the result was often unfavourable. The infants' and children's wards of which I had charge for some time were very nice, and the native babies were lovely little creatures, so delicately formed and so sweet to nurse. It was pathetic to see these small creatures suffering from plague and patiently bearing the suffering an older person might have rebelled against. Poor little things! some were only six months old, and it is wonderful how good they were and how soon they took to English treatment, quickly understanding the use of a feeding-bottle, which to their mothers' eyes was a novelty to be held in awe. The little cots were very crudely built up of pieces of old boxes.

When the plague had diminished so considerably in Poona that the nursing staff was reduced, four of the nurses, including myself, received orders to proceed to Bombay, where the epidemic was still at its height and the natives were in a state of revolt. They objected strongly to having their houses invaded and to being examined and segregated, and as they are so particular about their women being kept in strictest seclusion, any attempt to see them or to have them examined for plague symptoms was regarded with the greatest abhorrence; but, of course, it was absolutely necessary to do it. Ultimately the natives resorted to very strong measures and cruelly murdered two unprotected soldiers whom they recognised as being members of a search party. Things began to look most serious. They collected in large menacing crowds with great thick sticks, and seemed determined to break into the hospitals or else to burn them down. It was only when a detachment of soldiers arrived that the infuriated mob dispersed, and in order to keep peace in Grant Road, which is a thickly-populated native street in Bombay, guns were placed each end, facing down the street, ready to be fired any moment should the people show further signs of doing mischief. At Grant Road Hospital there was quite a panic, and one of our English doctors had to keep the crowd at bay with just one revolver until military aid arrived. Several members of the Plague Committee and other officials were soon on the spot, however, and an order was given to remove all the English nurses temporarily from the hospital. Accordingly, with an armed escort, they were conducted safely home until things were quieter, and the hospitals were left in charge of the doctors and ward assistants for 24 hours. They did all that was necessary for the patients. The nurses who were on duty at Grant Road Hospital had to live rather near the native quarters, and until things were quite secure and peaceful again they were always conducted backwards and forwards to the hospital under a military guard. In fact, all the plague hospitals in India were so protected for fear of any disturbance. On the morning after our arrival at Bombay the papers were full of the late riots, and things appeared very unsettled. Englishmen went about with revolvers and guns after dark, and it was unsafe to be out alone and unprotected, as the natives made many attempts to do their worst. About this time there was also a serious fire, owing to the carelessness of some of the servants. The European camp was burnt, and as the wards were made entirely of wood and thatch there was very little of it left standing in a short time. To add to the difficulties of the situation, some hay-ricks also caught fire, and these further carried the flames to the native plague camp, which was built on the other side of the road; and very soon the European and part of the native camp were both blazing. Every attempt was made to save the poor, helpless patients, and, happily, they all miraculously escaped being burnt, but many suffered afterwards seriously from shock. The nurses who occupied a tent in the camp had everything burnt but the clothes which they wore. This was a very great loss to them. However, people were most kind, and numerous things were sent to them for which they were very thankful, and were glad to wear them until they could get others made. Government also compensated them financially for their loss.

### Appointments.

THE ISLE OF WIGHT UNION.—On July 13th Miss C. M. Carr was appointed Assistant Matron and Midwifery Nurse. She was trained for three years at the Oldham Union Infirmary, held the post as sister for one year at the same place, and was private nurse for six months at the Zella Nursing Institute, Southport. At the present time she holds the post of charge night nurse at the Barnsley Union Infirmary.

JOHNSON HOSPITAL, SPALDING.—On July 17th Miss A. T. Wiginton was appointed Matron. She was trained at South Devon and East Cornwall Hospital, Plymouth, and she has since been matron of Yeatman Hospital, Sherborne, and in charge of a medical and surgical home.

### The Opening of Queen Charlotte's Nurses' Home.

THE ceremony of declaring open the new Nurses' Home attached to Queen Charlotte's Hospital was performed on Monday by the Duchess of York. Her Royal Highness, who was accompanied by the Duke of York, looked exceedingly well in a dress of black lace, trimmed with black and silver sequins, and a toque to match, brightened by a touch of heliotrope. They were received by Viscount Portman (president of the hospital) and the Countess of Leven and Melville, and after taking their places on the daïs a bouquet of purple orchids was presented to the Duchess by Master Gerard Street. Viscount PORTMAN then read an address welcoming the Royal guests and giving a sketch of the history of the hospital and of the present developments.

The Duke of YORK, in thanking Lord Portman and the authorities for the welcome accorded to the Duchess and himself, said that it afforded them great pleasure to be asked to take part in the interesting ceremony, more especially as so many of their family had shown their interest in the welfare of the charity. He congratulated the authorities upon the successful completion of the new home, and spoke of the need there was in such a hospital for a home for the many nurses who were being thoroughly and efficiently trained in the important and oftentimes difficult work they were called upon to perform. He understood that the sum of £7,000 was still needed, and he trusted that the amount would soon be raised. In conclusion, on behalf of the Duchess, he declared the Nurses' Home open.

After a short and appropriate service the Duchess of York accepted purses from ladies and children in aid of the building fund.

The Earl of HARDWICKE said the committee were cheered by the presence of the Duke and Duchess of York, but he emphasised the fact that they had still the bill to pay. The public must not think that the hospital authorities had been extravagant or reckless. There were investments that would enable them to meet their liabilities, but as the interest on these moneys helped to maintain the hospital, they had preferred to appeal to the generosity of the public rather than to encroach upon them. He was not going to harrow their feelings—that was the office of the right reverend prelate who would follow him. He had, on the contrary, a pleasing announcement to make. The Duchess of York had graciously consented that one ward should bear the name of the late Duchess of Teck and another her own. The fact that one ward was called the "Mary Adelaide" ward could not make the memory of that lamented princess more cherished and revered; and the fact that another would be known as the "May" ward could not deepen the love and affection all bore towards the Duchess of York; but those names would be a constant witness to those who suffered in those wards of the far-reaching sympathy and affection with which the Royal family regard all who are sick and suffering.

The BISHOP of LONDON did not harrow the feelings of the audience, but said only a few words emphasising the requirements of workers in the nursing field for places of rest and retirement. He also bore testimony to the value of such charities to those engaged in rescue work.

The Royal party then withdrew, after inspecting the home and the hospital.

The amount contained in the purses presented realised upwards of £250.

### Russian Famine Fund.

MR. J. T. WOOLRYCH PEROWNE writes from 3, Bryanston Place, W., to acknowledge the receipt of several more subscriptions through the "Nursing Mirror" to this fund.

### The Nursing of Sick Prisoners in Scottish Prisons.

### IN THE NAME OF OUR COMMON HUMANITY.

It will be recollected that a week or two ago we alluded to some important questions to be asked in the House of Commons by Mr. Parker Smith respecting the nursing of sick prisoners in Duke Street Prison, Glasgow. Receiving extremely unsatisfactory replies from the Lord Advocate, the member for the Partick Division of Lanarkshire intended last Friday to raise the matter on the Estimates, and we therefore deferred any comments upon it. But though he was prepared to bring it forward, other things prevented him from having the opportunity, and there is now no prospect of a discussion this session. Mr. Parker Smith was, indeed, able to obtain from the Lord Advocate, in reply to a question on Friday, an assurance that the Secretary for Scotland would investigate any specific case brought before his notice; but he intimated that the proposal for a general inquiry could not be entertained.

Nevertheless, the general inquiry is essential, and will, we trust, be pressed for until it is conceded. There are several powerful, not to say irresistible, arguments in favour of it. The first is that there has already been a searching inquiry into the treatment of prisoners, including, of course, sick prisoners, in England and Ireland. During the progress of that inquiry Sir Godfrey Lushington made a statement which supplies another good reason for action in regard to Scotland. Here is a quotation from his evidence: "Prisons are, and must be," he said, "dark places. Suggestions do not come pouring in as in cases of mines or factories. From prisoners you learn nothing. From warders, who are mere subordinates, and have to obey all orders strictly, you learn little more. Superior officers, of course, have greater knowledge, and might make useful recommendations, but I am sure they would think twice and three times before they ventured to volunteer to a strong minded and strong-willed executive, suggestions of a large kind which would involve an important alteration of machinery or a serious addition to the expense. So the suggestions came mainly from outside philanthropists who don't know the working." The complete helplessness of the prisoners to plead their own cause is a forcible argument why the suggestions of outside philanthropists should not necessarily fall on deaf ears. People who have broken the laws of their country may not be entitled to sympathy while they are strong and well; but, after all, they are our fellow creatures, and when they are sick they have indefeasible claims to care and attention. This is precisely what is refused to them in Scottish prisons under existing conditions.

The cases to which Mr. Parker Smith called attention, with the result that his facts were admitted, would alone suffice to justify our assertion. In that of Mary Carroll, the Lord Advocate did not deny that the woman, who was suffering from gangrene of the foot, was taken during the night from her solitary cell, in what proved to be a dying condition, to an association cell already occupied by a sick prisoner in company of two other prisoners. Here she was left, and an hour or so after she died. The nurse warder who ordered her to be removed to the association cell clearly did not realise the grave condition of the prisoner; but no blame is to be attributed to her. Her knowledge is limited, and even if it had been otherwise she is at work all day, and ought not to be expected to discharge night duties also. With an average population of 400 women in Duke Street prison there is only one nurse warder. In other words, sick prisoners are treated in ordinary cells, and are left alone unnursed all night for want of proper hospital accommodation and an adequate hospital nursing staff.

The contention that the case of Mary Carroll is only typical derives indirect confirmation from the annual report of the Prison Commissioners for Scotland for last year. In all the prisons there were 1,865 cases of illness, of which no more than 16 were sent to the hospital, four of them dying shortly after removal. But 19 also died in prison from various causes, such as acute pneumonia, apoplexy, cardiac failure, bronchitis, and congestion. Another point which merits the attention of those who are disposed to lightly consider the sufferings of incorrigible offenders is that out of the 38,892 separate individuals confined in Scottish prisons in 1898 upwards of 17,000 had never been in prison before; and the report of the Commissioners says, "The slight criminal character of the offences of which the great majority of prisoners had been guilty is well brought out by the average length of the sentences imposed during the year. From the judicial statistics we find it was fifteen days." Yet these first offenders, guilty only of crimes punishable, perhaps, by almost nominal imprisonment, are, if sick, left to the tender mercies of their fellow-prisoners. Thus, at Ayr Gaol, to which from 2,000 to 3,000 prisoners are admitted annually, there is not a hospital warder of any kind for either males or females, and the nursing of the sick is entirely in the hands of the fellow-criminals "associated" with them for the purpose.

But the *crux* of the situation is that, supposing there were special warders in every prison on nursing duty, they are not sufficient for the purpose. They have not had proper training, and they are constantly being changed. At Duke Street Prison, Glasgow, and at many other Scottish prisons, the hospital accommodation is miserably inadequate, if not an absolute farce. A hospital ward usually seems to mean a couple of ordinary cells knocked into one. This is the first thing to remedy. The next is to substitute for specially selected prisoners, or nurse warders, trained nurses of the best class, who, as in hospitals or infirmaries, would devote their whole time to the task of nursing sick prisoners. That these results would follow a searching general inquiry, such as Mr. Parker Smith advocates, cannot be doubted. Why Scottish sick prisoners should be treated so much worse than English and Irish is unintelligible; and in the name of our common humanity we appeal to the Secretary for Scotland to take the initial step with the view of bringing about a reform in a condition of affairs which is condemned by public opinion, and is contrary to the elementary principles of Christianity. It is an extraordinary reflection even on our boasted civilisation, that at the close of the nineteenth century it should be possible for a Minister of the Crown to state, without the suggestion of an apology, that the arrangements in any prison in the Queen's dominions are of such a character as to allow of a sick prisoner dying unnursed and unattended by any responsible person. The Scottish people owe it to themselves to drive the nail home without delay.

### To Nurses.

In order to increase and vary the interest in the *Mirror*, we invite contributions from any of our readers in the form of either an article, a paragraph, or information, and will pay a minimum of 5s. for each contribution. All rejected manuscripts are returned in due course, and all payments for manuscripts used are made at the beginning of each quarter, *i.e.*, January 1st, April 1st, July 1st, and October 1st.

### Examination Questions for Nurses.

NURSE WARBURTON is the winner of the first prize, and Nurse Parsons of the second. The competition has been much more satisfactory this month, and a large proportion of excellent papers have been sent in. As the question combined four sections there is some difficulty in choosing the paper that should deal best with all the different points. Nurse Warburton has dealt best with the two middle questions concerning the manufacture of a mustard and of a charcoal poultice. Several candidates mistake the difference between a plaster and a poultice, which are indeed two completely different things; one confounds mustard and charcoal together in one fell compound, and gives these remarkable instructions: "Mix mustard with chilled water, adding the required quantity of charcoal, and apply it sandwiched between brown paper"!

Some others speak of mixing the dry mustard and linseed together before sprinkling into the water; this is always a mistake, because there would be small unmixed portions of mustard very liable to raise a blister. The mustard must be first mixed in nearly boiling water to avoid this danger. Another candidate boldly says: "Keep it on till it raises a blister"! I should be sorry to be nursed by these doubtless well-intentioned but ill-trained persons.

Nurse Warburton has not worded her paper well, so that the hasty reader will hardly understand where the directions for making a jacket poultice begin, but the matter is good if the manner lacks something. I would, however, earnestly recommend all candidates to try to make their explanations thoroughly intelligible, and their writing good and neat. We shall resume these questions in the late autumn.

### FIRST PRIZE.

To make a simple poultice of any use you must have the water boiling and the bowl and knife or spatula warmed. Only stir in sufficient meal to make the mixture thick enough to spread without sticking to the knife. Turn in the edges of poultice all round to keep in the heat, and don't let it be too small or out of proportion in thickness. Roll up poultice in a flannel or put between hot plates to carry to bedside, and apply without delay, with a piece of thin mackintosh a little larger than poultice next it, and wadding or flannel outside of all. Fasten securely with bandage or binder on the part needing poultice, and when removing wash the part with warm water and dry before replacing with hot flannel or wadding, or, if repeating poultice, have the fresh one ready before removing the old one, so as not to chill the patient. Paper or flannel may be used, failing linen, or tow well teased and pulled out. For a mustard poultice blend the desired quantity of mustard (usually one in four of linseed) in the water before stirring in the linseed meal and proceed as before. For a charcoal poultice mix the charcoal (one part to three of linseed) with the meal first, then stir into the boiling water, proceeding as before. Two large pieces of linen should be shaped out, one for the chest and one for the back and sides. After the poultices are applied the edges are secured over the shoulders and under the arms with pins, so as to form a jacket. The simplest way of securing a jacket is a binder made of a broad strip of flannel or flannelette to go round the body, and two straps sewed on behind on its upper edge, passing over the shoulders and pinned in front. When a patient cannot sit up he can be turned on one side, the back poultice made and applied, the binder with one end rolled placed in position; the patient is then turned on his back lying on the poultice, while the rolled end of binder is drawn round, the chest poultice made and applied, and all secured with safety pins.

### SECOND PRIZE.

(1) The linen should be large enough to allow a one-inch turning over the edge of the poultice, which should be made of linseed meal and applied to the skin as hot as it can be borne. A little olive oil spread on the surface cools it, and enables the patient to bear it hotter; it also prevents sticking, and you are less likely to get a poultice rash. Cover with waterproof, cotton wool, and bandage securely. Omit the waterproof when poulticing surgical cases.

(2) Failing linen, I should use tow, which should be well teased out, crossed and recrossed, till you get a groundwork sufficiently thick. Waterproof paper is useful, and serves two purposes; thick brown paper, or useless bits of rag sewn together.

(3) Mustard poultice.—To one pound of meal use two heaped tablespoons of mustard. First mix the mustard with a little water till perfectly smooth. This allows the mustard being equally distributed amongst the meal, and also prevents blisters being formed by little lumps of mustard being in contact with the skin a long time. Then put in the boiling water and stir in the meal with a hot spatula. Too much water must not be put into the basin, or the meal will be out of proportion to the mustard to make it the required consistency. This poultice must be watched in case of blistering delicate skins.

Charcoal Poultice.—One part of charcoal to two parts of meal. First mix them together dry, then make in the usual way.

(4.) A jacket poultice is a large poultice to cover the chest and back, made in two halves, and meeting under each arm and over the shoulders. It is best fastened securely by safety pins down the sides and over the shoulders, covered with thin mackintosh and cotton wool. The whole is bound on to the body by passing round the body a long, straight piece of flannel in which has been cut two straight slits to allow of its passing under the arms, across the chest, and over the shoulders, to be pinned to the flannel at the back. The linen on which the poultice is made should be shaped out like a vest—hollowed out at the neck and armpits. The patient should be undressed and turned on to his side before the poultice is made, and that for the back applied first, covered with mackintosh and wool, and tucked as far as possible under the arm on which he lies; then cover with flannel, passing one slit under the free arm and one as far as possible under the other side. He must be turned on to his back while the chest is poulticed. The two poultices are then pinned together down the sides and shoulders, covered with wool, and the two ends of flannel crossed over each other on the chest and pinned, the flannel at the back of the neck being brought over the shoulders and pinned in front.

I think it merciful to let the patient remain on his side while I prepare the chest poultice, rather than bring him back at once to lie on the stinging hot mass.

### The London Hospital.

### ANNUAL DISTRIBUTION OF PRIZES TO STUDENTS AND PROBATIONERS.

TUESDAY was a great day at the London Hospital, combining two events of importance, namely, the opening of the new Medical College Buildings, and the annual distribution of prizes to students and probationers. The latter is always a pleasant meeting time for past and present Londoners, a large proportion of the former invariably putting in an appearance on such occasions. The library of the college was filled to overflowing for the ceremony, and was very prettily decorated with palms and plants. Lady Knutsford made a pleasant little speech to the nurses before presenting the prizes and certificates to those fortunate probationers who had earned this coveted honour. The three prizes, £5 5s., £4 4s., and £3 3s. respectively, were awarded to Probationers Ethel Bamford, Isabella K. Grant, and Ethel Pearson; and honorary certificates were presented to Probationers Minchim, Harper, Sleight, Dickenson, Adair, Dent, and Roberts. Refreshments were served in the garden, and the lawn was a gay sight when the visitors trouped out of the college and spread themselves over its wide expanse, to be made happy with tea and ices, before making the tour of wards and the nurses' home, not to mention the "Garden of Eden," and the church, in which all the staff of the hospital take such pleasure and pride. Miss Lückes had a busy time with so many guests, and the "old workers," who all want a word with "Matron" before they go, but everyone had their greeting. Amongst others present were Miss Bland, Miss Cave, Mrs. F. Smith, Miss Rowe, Mrs. J. Hutchinson, and Miss Lamport.

### Echoes from the Outside World.

### AN OPEN LETTER TO A HOSPITAL NURSE.

It is not often that I tell you about a wedding, but the marriage of Lady Constance Grosvenor and the Earl of Shaftesbury was such an uncommonly pretty function that I think you might like to hear a little about it. The bride, a very sweet girl, is the elder daughter of the Countess Grosvenor, and granddaughter of the Duke of Westminster, and she looked exceedingly nice in her beautiful wedding gown. It was of very rich white satin, bordered with silver lace and pearl embroidery, opening over a petticoat of frilled chiffon. The bodice had a Brussels lace collar and yoke and the dearest little coat, or zouave, of the same lovely fabric. Her lace veil was also of Brussels, and was, I am told, a family heirloom. It was arranged over quite a coronet of orange blossoms. The bridesmaids seemed never ending, and no wonder, for there were fifteen in all, five of whom were children. Their dresses looked cool and charming, white spotted, or rather sprigged, net over white satin, with shaped skirts draped round the bottom. The bodices had fichus most daintily arranged, with a big bunch of the bride's favourite flowers—which are called Roses de Bengale, I believe, but remind one of the old-fashioned pink china roses we all love—placed on the left side and tied together with narrow blue satin moiré ribbon, which hung with long ends in front. The same blue ribbon also encircled the waist. The hats were of drawn white chiffon, with touches of the pale blue ribbon, a wreath of myrtle on the brim, and big upstanding bunches of the roses, which predominated also in the bouquets. I heard someone whisper that the roses had been specially grown for the purpose at Eaton Hall, but I could scarcely credit that a single garden could produce such a number, so I took the rumour for what it was worth. The bridegroom's gift to the maids was unique—an oval slide medallion formed of a spray of forget-me-nots in blue enamel, with leaves in diamonds. It was worn round the neck, threaded with a white ribbon. There were over a thousand presents, eight hundred of which were for the bride.

The fact that the Prime Minister's family always make the least, instead of the most, of any case of indisposition among them, invests with all the greater gravity the illness of the Marchioness of Salisbury. Only the most serious anxiety on account of his wife would have caused Lord Salisbury to remain at Walmer or to abandon the engagement to dine with the members of the Liberal Union Club on Wednesday evening. The latest accounts are more reassuring, but the sympathy of the nation goes out to the illustrious statesman who has always found in his own home the sunshine of his life. The generous but well-deserved tribute which has been paid to him and to Lady Salisbury by the leading organ of the Opposition is one of many indications of the regard in which they are held, not merely for their rank and eminence, but for their character—the one thing that never fails to impress all sorts and conditions of people. I am quite sure that all of you will join in the hope that Lady Salisbury may be spared for many years to share her husband's cares and joys.

If it were not so near the end of the season, I am afraid that the example set by Lady Doreen Long and Lady Frances Balfour on the occasion of the passage of the Clergy Rating Bill through Committee in the House of Commons would be followed at once, and it would become quite the fashion to go down to the House and stay until four a.m. Of course, the hour is no later than that at which fashionable balls occasionally end, and the idea of being able to enjoy quite an original sort of entertainment, including suppers of devilled bones and other luxuries, lounges on the terrace beside the gleaming river, with the stars overhead and the cool breeze playing around, followed by choice little early breakfasts later on in the dining-room before returning home, sounds very fascinating. This is more especially the case when the ladies have the additional satisfaction of feeling that, as well as amusing themselves, they are showing their devotion to their party and their men-kind.

I am glad to see an appeal from the Society for the Prevention of Cruelty to Animals on behalf of the vacation cats. It has always seemed to me, in a small way, one of the most diabolical acts for a woman to go away to the seaside or the Continent for a month or six weeks, knowing she would probably have a good time herself, and simply to turn her cat out into the streets to starve, or to thieve for itself a bare existence, resulting later on very likely in the development of disease. I am certain that if I did such an inhuman deed I should be haunted by the nightmare of a fearful Grimalkin with bones sticking out through its flesh, and with big, hungry eyes, stalking me down in just retribution. Surely if it is not always feasible to leave a caretaker in the house, the good offices of some kindly-disposed neighbour might be called into requisition, who would put out some milk and food for poor Pussy at regular intervals. I believe that for a trifling payment there are many private individuals as well as institutions who will receive temporarily homeless cats, and perhaps it would be a good thing if the Servants' Registry Offices would keep a list of these folks, who, for a consideration, would act the good Samaritan to the so-called "family pet." Whether or not the cat would always consent to stop with a stranger is another matter.

The idea of an English musician composing a famous opera is always treated with so much incredulity and contempt by musical critics that when a composition by a native of England—even though he boasts an Italian name—is considered worthy of a place in Covent Garden the fact must not go unrecorded. The second performance of "Messaline" took place on Wednesday, and the good impression as to the merits of Mr. Isidore de Lara's work which had been made last week was confirmed. One cannot help regretting that the plot of the play should be so exceedingly disagreeable, but apparently, for some strange reason, few musicians can become inspired to write grand harmonies unless they have an immoral theme to help them. Also on the first evening the superabundance of the kissing element was a little oppressive. Not only did the characters keep on singing about the delights of osculation, but they frequently, most frequently, put it into practice. The opera is in four acts, the second especially containing some beautiful music, while the last, both from a dramatic and musical standpoint, is wonderfully strong. The orchestration is at all times very suggestive, more particularly in the grand dark music representative of Death in the gladiator scene. Madame Héglon, a native of Brussels, who married at seventeen and lost her husband a year after, and then devoted herself to the operatic stage, is most capable in the rôle of the Empress; her acting is overwhelmingly passionate, and her voice is magnificent.

Because less has been heard lately about the wholesale slaughter of birds in order to obtain egret feathers for fashionable dames, we had begun to hope that the trouble had past, and I for one quite believed the milliners who met objections to the use of the delicate white sprays by the smiling assurance that they were not the "real" thing—they were all now made artificially. But, unfortunately, it appears that this has been a dodge on the part of the shops to lull the scruples of their customers and persuade them to go on paying for egrets. The Consular report on the trade of Venezuela shows that last year more than a million and a-half of birds were slaughtered in that country alone to decorate the hats of so-called Christian women!

### Everybody's Opinion.

[Correspondence on all subjects is invited, but we cannot in any way be responsible for the opinions expressed by our correspondents. No communication can be entertained if the name and address of the correspondent is not given, as a guarantee of good faith but not necessarily for publication, or unless one side of the paper only is written on.]

### CATERPILLAR RASH.

"The Matron of the Convalescent Home at Wotton-under-Edge" writes: In answer to the inquiry of "Nurse Matron," a description of a caterpillar producing much the same condition of the skin is to be found in "Common Objects of the Country," by the Rev. C. A. Wood. The caterpillar, I believe, is a small hairy one, and, I suppose, has some fluid as a weapon of defence when handled. It is years since I was a collector of insects, but I quite remember the irritation caused by this caterpillar.

### HELPS TO TRAINED SISTERS.

"The Hon. Local Secretary of the Ipswich Centre of the St. John Ambulance Association" writes: In the "Nursing Mirror" of July 8th I read of what German ladies do in connection with the Order of St. John of Jerusalem. Did no English lady rise at the Congress and say we do much the same in connection with the same Order? Miss Topping, of St. John's House, Worcester, is a trained nurse of Guy's Hospital. The home is constantly full of ladies taking a course of practical nursing. The home was established seventeen years ago. It seems strange that no one knew of this.

### PAYING v. NON-PAYING PROBATIONERS.

"A. M. M." writes: I have been thinking over the suggestion which has been made that only paying probationers should be allowed to enter for training in the hospitals. If brains and money always went together, of course the proposal would be a good one. But as it is I do not think the scheme is ever likely to be realised, nor do I fear that the bees who have to work so hard for every bit of sweetness which they enjoy through life need be in the least alarmed at the squeal of the "guinea pig" now or at any time. Supposing such a thing to be possible, and that the London hospitals were to close their doors against non-paying probationers, what an excitement there would be amongst the nurses. Our voices would be heard in the land without a doubt. I have had my training, which I did not pay for, so have no need to think of myself, but I speak for others who are coming on and may be placed in similar circumstances.

### PRIVATE NURSING INSTITUTIONS.

Nurse Mitchell writes: After reading several of your letters as to "Why nurses object to joining nursing institutions," I would like to give a slight sketch of my experience in a well-known establishment. I joined the home with a seven years' reference from one of the largest London infirmaries. They gave me a salary at this home of £30 per annum, telling me it did not pay them to give more. However, I was sent to fever work all the time, for which they made a charge of 30s. per week, and 30s. quarantine fee at finish of case. (This extra fee, by their printed forms sent to patients, is required for disinfection, and so that the nurse shall not be required to go out for a week.) But they claim the entire 30s., and do not even give us our fare to the small cottage used elsewhere for fever nurses. Also all the time I was there I bought my own disinfectants; they never asked if my things were cleansed, and more often than otherwise we were sent right off, or only with a couple of days' quarantine, to other cases, after nursing typhoid, scarlet, and diphtheria. The last eight weeks I spent at a small scarlet fever hospital. On April 4th I was paid my quarter's salary. My brother was taken ill, but as I was still on duty I gave up my only chance of seeing him because of the infection and so as not to inconvenience them at the home. Once I had to telephone constantly for three days before they sent me a nurse out to help me when one had gone back, and I was left completely alone with eight children. Then I was called home at short notice. I sent them word that I must return as soon as possible. This was on May 4th; I left one week later. But previously I wrote two letters to the home requesting someone to see me on Wednesday morning on my way to the station and settle with me. I called to get my luggage and receive, as I thought, at least some of my five weeks' salary. They refused to see me. I came on to London, and after writing twice Mrs. —— came to my friend's house in London, and I told her I expected her to pay me the week, even if she chose to keep the month's salary, and that unless she settled I should see a solicitor; but considering that I had been working hard for her all the time, and giving her a week's notice when I badly wanted to be elsewhere, I thought she might give me a little more. Then there was my quarantine 30s., which they drew after I had left their establishment altogether. Well, she finished by rushing out of the house and refusing to pay me anything whatever. After again writing, I waited another fortnight before my solicitor wrote, when they sent me 12s. as five weeks' pay, for which they drew £7 10s. and 30s. for quarantine, in all £9.

### Minor Appointments.

Middlesbrough Union.—On July 13th Miss A. L. Lench was appointed Charge-Nurse of the Workhouse Infirmary. She was trained at the Workhouse Hospital, Carlisle. She has since been nurse at the Workhouse Hospital at Wigton, and charge-nurse successively at the Workhouse Hospitals, Stoke-on-Trent, Haslingden, and Todmorden.

Brighton Workhouse.—On July 11th Miss Isabel H. Myles was appointed Staff Nurse. She was trained at the Infirmary, Birmingham, and she has since been sister of the male surgical ward and home sister of the Workhouse Infirmary, Birmingham.

Chelsea Infirmary.—Miss Evelyn Dawson has been appointed Night-Superintendent. Miss Dawson was trained at St. Bartholomew's Hospital.

### Presentations.

The Wolverhampton General Hospital.—Last week Mrs. George White, who has been matron of the Wolverhampton General Hospital for more than fifteen years, was presented by the nurses and resident staff with a handsome silver tea service, to which the domestic staff added a very beautiful inlaid Chippendale tray. The presentation was made in the name of the subscribers by the Rev. J. Warner, chaplain of the hospital, who referred in a very brief speech to the long period during which Mrs. White had been matron of the hospital, and to the kindly feeling which had prompted the nurses, as well as many who had formerly been attached to the nursing staff and had received their training under the present matron, to subscribe to the gift. Mrs. White was also the recipient of a cheque for £70, subscribed by members of the board of management, members of the honorary staff, and other friends.

Royal Infirmary, Hull.—Miss A. K. Brooks, on leaving the Royal Infirmary, Hull, to take up her new appointment as assistant matron of St. Mary's Hospital, Manchester, has been presented by the infirmary staff with a silver teapot, cream jug, and sugar basin. Miss Brooks has been on the staff for eight and a-half years, and her departure is much regretted by all.

### Where to Go.

Nurses who are going to Marlborough House on Friday will be glad to know that the Trained Nurses' Club, 12, Buckingham Street, will be open all day for the convenience of those who like to make use of it. A cup of tea and a little rest in the coolness of the club rooms will be a help to many no doubt, and Miss Clinton will welcome visitors and send them on their way refreshed.

### For Reading to the Sick.

"TASTE and see that the Lord is sweet. Blessed is the man that hopeth in Him."

Hopes of every sort,—whatever sect
Esteem them, sow them, rear them, and protect,
If wild the nature and not duly found,
Gethsemane! in thy dear, hallowed ground—
That cannot bear the blaze of Scripture light,
Nor cheer the spirit, nor refresh the sight,
Nor animate the soul to Christian deeds,—
(Oh, cast them from thee!) are weeds, arrant weeds.
—*Cowper.*

When first Thy sweet and gracious eye
Vouchsafed e'en in the midst of youth and night
To look upon me, who before did lie
Weltering in sin,
I felt a sugared, strange delight,
Passing all cordials made by any art,
Bedew, embalm, and overrun my heart,
And take it in.
Since that time many a bitter storm
My soul hath felt, e'en able to destroy,
Had the malicious and ill-meaning harm
His swing and sway;
But still Thy sweet original joy
Sprung from Thine eye, did work within my soul,
And surging griefs, when they grow bold, control
And get the day.
If Thy first glance so powerful be
Mirth but opened and sealed up again,
What wonders shall we feel when we shall see
Thy full-eyed love!
When Thou shalt look us out of pain,
And one aspect of Thine spend in delight
More than a thousand suns disburse in light
In Heaven above.—*Herbert.*

Longing is God's fresh heavenward will
With our poor earthward striving;
We quench it that we may be still
Content with merely living.
But would we learn the heart's full scope,
Which we are hourly wronging,
Our lives must climb from hope to hope
And realise our longing! —*Lowell.*

As reason is a gift of God in the order of nature, so faith is a gift of God in the order of grace; and as the order of grace is higher and better than the order of nature, so faith is higher and better than any natural gift. Both reason and faith may be lost. But it were better far to lose even our natural life than to lose our supernatural faith. . . . All our lives we are making trial by experience of the goodness of God. You have known it from your childhood. You have known it by the manifold and multiplied indications of His love to you in every period of your life, by the care with which He has watched over you, by all the operations of grace whereby He has guided you, all the absolutions He has bestowed on you, by the peace he has shed abroad in your heart, by the help he has given you in temptation, by the consolations that have come down upon you like showers in the time of your sorrow and desolation of heart. All this has taught you to know Him, and to say out of the depth of your own experience what the Psalmist said, "I believe that I shall see the good things of the Lord in the land of the living," and again when he said, "Taste and see that the Lord is sweet. Blessed is the man that hopeth in Him." "How great is the multitude of Thy sweetness, which Thou hast hidden for them that fear thee." Such then is the nature of hope.

### Notes and Queries.

**The contents of the Editor's Letter-box have now reached such unwieldy proportions that it has become necessary to establish a hard and fast rule regarding Answers to Correspondents. In future, all questions requiring replies will continue to be answered in this column without any fee. If an answer is required by letter, a fee of half-a-crown must be enclosed with the note containing the enquiry. We are always pleased to help our numerous correspondents to the fullest extent, and we can trust them to sympathise in the overwhelming amount of writing which makes the new rules a necessity.**

**Every communication must be accompanied by the writer's name and address, otherwise it will receive no attention.**

*Idiot Child.*

(138) Can you tell me of a home or institute in or near London where an idiot boy, aged six, who can neither speak or walk, could be taken care of? The parents could pay a little towards maintenance.—*Nurse E. C.*

The Earlswood Asylum for Idiots and Imbeciles at Redhill would be the best. Admission is by annual payment or by election of subscriber. The address of office is 36, King William Street, E.C.

*Probationer of 18 Yea s.*

(139) Will you help me by giving me the particulars required concerning my entering a hospital or infirmary as a probationer at the age of 18 years?—*M. S.*

See "The Nursing Profession: How and Where to Train" (2s., the Scientific Press, London, W.) In it you will find full particulars of all the training schools. You will also see that there are only one or two children's hospitals which will accept candidates so young as the age you mention.

*Probationer.*

(140) Would you kindly tell me the address of a hospital in London where a lady could be trained as nurse, where the work would not be too hard, and where one could get knowledge of nursing after a year or two's training? I am a probationer at present, and I find the work almost killing. Moreover, it goes on from week to week, and I find it is still the same after two years. The nurses are bound here for four years. If I could, I should like to get into a private or cottage hospital, where I should not have such hard work, and where explanations are given.—*Nurse.*

You have evidently been unfortunate in your choice of a training school—perhaps in your choice of a profession. Make sure, before you continue nursing, that you are strong enough for it. It is killing work, and none but the strongest are fit for it. "The Nursing Profession: How and Where to Train" (2s., Scientific Press) gives particulars of the different training schools, and would enable you to make a more advantageous selection.

*Massage.*

(141) Will you kindly let me know if any of the hospital training schools in London will give a special course in massage to a graduate nurse of another school; if so, length of time required and cost? Are there any schools exclusively for massage you could recommend? I hope to visit London next year, and wish to perfect myself in that branch.—*America.*

None of the London hospitals' training schools give post graduate teaching, but there are plenty of schools for instruction in massage. You will find advertisements of reliable establishments in the "Mirror," and the Society of Trained Masseuses, 12, Buckingham Street, Strand, W.C., would be able to advise you in the matter.

*Examination Questions.*

(142) Will you kindly tell me what will be the correct address to use if I forward an answer to the July "Question" in the "Nursing Mirror"? There was something to be written in the lefthand corner of the envelope which I have forgotten.—*District Nurse.*

In the lefthand corner write the words "Examination Question," and address the Editor, the "Nursing Mirror."

*Cycling.*

(143) Can you give me your opinion with regard to ladies cycling? Is it really beneficial to some and not to others? Or why is it specialists are so prejudiced against it, and yet many doctors advocate it.—*A. P.*

It is generally admitted that the moderate use of the bicycle is beneficial to most women. Some, however, for physical reasons, either temporary or permanent, are debarred from taking part in it. Are specialists prejudiced against it? And, if so, in what are they specialists?

*Birmingham Infirmary.*

(144) Will you kindly say if the Birmingham Workhouse Infirmary is a good training school? Also, how many beds there are at the same?—*C. T.*

The Birmingham Infirmary is an important training school. The number of beds is 1,540.

*Diphtheria.*

(145) Will you kindly tell me what is done with regard to fee, &c., f a nurse contracts diphtheria from her patient in a private house a long distance from a fever hospital? Would the nurse forfeit her fee, and be responsible for any expense incurred by her illness?—*Sister Mon ca.*

This is one of the risks that a private nurse must face. Under such circumstances she forfeits her fee and must bear the expense of her illness and convalescence.

*Outfit for South Africa.*

(146) I should be very glad if you will give me advice as to what outfit is necessary for a nurse going out to South Africa. My brother has been ordered there for his health, and I am to accompany him, but we do not yet know to what part we are to go, except that it must be inland —*April.*

Until you know the part of South Africa to which you are going, it is difficult to advise you. The inland climate is sometimes quite cold.

### Travel Notes.

By Sister Grace.

### XXIX.—KONIGSWINTER ON THE RHINE.

A fortnight spent in breezily flying up and down the Rhine either on a bicycle, your own legs, or on one of the many steamers that ply up and down the mighty river, makes a delightful holiday, and one that will suit the pockets of most nurses, for the expenses may be limited to seven or eight pounds.

### The Journey.

The return fare to Bonn, second-class, is £2 7s. 9d. You leave Liverpool Street at 8.30 p.m., reach the Hook at 5 in the morning, go on by rail to Cologne at 5.33 a.m., which is reached at midday. You will get out and see the marvellous cathedral, of course; it is close to the station, and there will be ample time, for a train goes on conveniently at 2 p.m. Your luggage need give you no anxiety, it will have been registered through and will await you at Bonn, where you will take to the steamer and go up to Konigswinter, or if economy is very important it is cheaper to go the entire way by rail. As you will be up and down the river continually you need not grudge the loss of the steamer transit on this occasion.

### Accommodation.

At Konigswinter go to the Hotel Reiffel, pension terms five marks, or to the Hotel Bockhalle, Haupt Strasse, four marks. These hotels suit slender purses, but if something more luxurious is desired try the Hotel Mattern with outlook to the river.

### The Drachenfels, &c.

Konigswinter is the best spot for visiting the Drachenfels and Seven Mountains generally, of which one obtains the first peep at Cologne; on a nearer approach some of them are obscured. There is now a rack and pinion railway to the summit of the Drachenfels, which is sad desecration, but the flesh is weak, and one must admit that there are extenuating circumstances attendant on the atrocity. Poor walkers and those not very strong can now enjoy the magnificent scenery without fatigue. The ascent is of ten minutes' duration, fare one mark. Remember a mark is practically of the same value as a shilling. To walk takes three-quarters of an hour, and if you are a good pedestrian it is a real treat, preferable to any other way. The Siebengebirge (seven mountains) may be almost entirely visited by carriage now, but going on foot and taking one's time is far more enjoyable. A good plan for fair walkers is to take the mountain railway to the summit of the Drachenfels, so as to be fresh to cross to the Great Oelberg, which takes an hour and three-quarters. This mountain commands one of the most magnificent views of the neighbourhood, and the whole excursion is one of unsurpassed loveliness. After a rest go on to Heisterbach, time occupied one hour and a-quarter. After that another three-quarters of an hour brings you back to your hotel. It is by no means a very fatiguing walk if you allow yourself plenty of time, and one which must give pleasure.

### The Neighbourhood.

If you are cyclists you will be able to explore the river up to Coblentz. It is better to "do" one part of the Rhine thoroughly one year, and not strive to see everything in one visit. If you devote a fortnight to seeing the scenery between Bonn and Coblentz, you will not leave much unexplored in that direction, and another year you can make your headquarters at a spot higher up. In front of Honnef, a short three miles from Konigswinter, are the romantic islands of Nonnenwerth and Grafenwerth, with Rolandseck opposite. Ten miles from Konigswinter is Linz, reached by cycle, steamer, or rail, and on the opposite bank Remagen, from which place you can make a lovely and cheap excursion up the valley of the Ahr by rail in two and a quarter hours to Adenau; return fare, 3 marks. It is an exquisite spot, and generally quite overlooked by those who hurry through the Rhine country and consider they know the mighty river after a week's acquaintance. Andernach is a very picturesque little town on the right bank, 20 miles on the way to Coblentz. As you gaze upon it it will seem familiar, because its fine old church and watch tower are such favourite subjects with artists. Many grand buildings have been destroyed in the constant wars, but much still remains, notably the Rathhaus. Between Bonn and Coblentz the river banks are studded with the remnants of splendid castles more or less in a state of decay, but space does not admit of my speaking of them. There are various cheap guide-books that will help you when you visit this delightful country.

### Hints to Travellers.

I feel an inward conviction that very few intending travellers will think to look back to the first weeks of December in last year, when these travel notes first appeared, and when I gave practical hints to those bent on foreign tours. I shall therefore from time to time repeat these remarks. From the questions put to me I see there is still much misconception on the burning questions of dress, luggage generally, and what to take and what to avoid.

---

### TRAVEL NOTES AND QUERIES.

Rules in Regard to Correspondence for this Section.—All questioners must use a pseudonym for publication, but the communication must also bear the writer's own name and address as well, which will be regarded as confidential. All such communications to be addressed "Travel Editor, 'Nursing Mirror,' 28, Southampton Street, Strand." No charge will be made for inserting and answering questions in the inquiry column, and all will be answered in rotation as space permits. If an answer by letter is required, a stamped and addressed envelope must be enclosed, together with 2s. 6d., which fee will be devoted to the objects of the "Hospital Convalescent Fund." Any inquiries reaching the office after Monday cannot be answered in "The Mirror" of the current week.

Genoa (Bicycle).—The climate is mild in winter and somewhat hot in summer, still the sea breezes temper the heat, and I spent a very hot September there without experiencing any inconvenience.

Landerneau (Duplex).—Landerneau and Lamballe are very taking places to the artist, but not to the general tourist. Both are out of the way places in Brittany, but being junctions are good places for excursions, but they do not seem to me to be quite what you want. How would the banks of the Loire suit?

Italian Lakes (Como).—May is the ideal month; as that is in the past, September comes next best. No, I should hardly call it good cycling country. Take warm clothing if you go in September.

France for Holiday and Education (Mrs. J. C.)—You give me no pseudonym, so can only hope this will meet your eye. Boarding in a French family for a short time is out of the question. French people are strongly averse to entertaining strangers. A moderate hotel or pension is your best chance, but at all cheap seaside places English abound unfortunately. You could board at from 6 to 7 frs., that is to say, from 5s. to 6s. per day each at Veules, reached by rail from St. Valery; it is on the Norman coast, about twenty miles from Dieppe; Fécamp, further on, about the same. I should recommend Paramé or St. Servan, in Brittany (articles in "Mirror" some weeks since), reached viâ Southampton. There is no railway journey on landing. When you have decided whether you prefer Normandy and Brittany I can give you more particulars. My advice is decidedly St. Servan or Paramé.

A Fortnight in Dinard or St. Servan (Mrs. A. L.)—You give me no pseudonym, so I use your initials. Dinard is out of the question on the amount you have to spend, but residence at St. Servan or Paramé may be managed for a fortnight with great care. Return ticket £1 19s. (a trifle less if you travel to Southampton), third class. I think I should recommend Paramé in preference to St. Servan in your case. Write to the proprietor of Hôtel des Bains and Hôtel du Centre, and ask if he would take you for 5.50 per day; he advertises at 6 frcs., but sometimes for a fortnight he will take less. A third for the same terms is Hôtel de l'Ocean. If 6 frcs., a fortnight would come to 84 frcs., tips, &c., to 100 frcs., making £4, this leaves you £3 for your journey and excursions. You will only be able to go to Mont St. Michael for the day if you do not exceed £7 each. It is imperatively necessary to belong to the C.T.C., address 47, Victoria Street, Westminster. With this you can take your cycles into France free. At the Custom House you will pay 60 centimes for a "Permis de circulation." You must cross to St. Malo by the night boat, there is no day service. Get your tickets through Messrs. Gaze and Son, 142, Strand. All particulars to be found of excursions in two articles published in the "Mirror" of May 6th and 13th.

## Figures and Tables

**Figure f1:**